# Intraprostatic Injection of Alcohol Gel for the Treatment of Benign Prostatic Hyperplasia: Preliminary Clinical Results

**DOI:** 10.1100/tsw.2007.385

**Published:** 2006-09-06

**Authors:** Benjamin T. Larson, Nelson Netto, Christian Huidobro, Marcelo Lopez de Lima, Wagner Matheus, Cristian Acevedo, Thayne R. Larson

**Affiliations:** ^1^ Institute of Medical Research, Scottsdale, Arizona, USA; ^2^ Cleveland Clinic Lerner College of Medicine of Case Western Reserve University, Cleveland, Ohio, USA; ^3^ University of Campinas, Faculdade de Ciencias Medicas-Unicamp, Sao Paulo, Brazil; ^4^ Department of Urology, University of Chile, Santiago, Chile

**Keywords:** alcohol gel, BPH, intraprostatic injection, minimally invasive

## Abstract

Benign prostatic hyperplasia (BPH) is one of the most common diseases ailing older men. Office-based procedures offer the advantage of being more effective than medications, while limiting the adverse effects, cost, and recovery of surgery. This study presents preliminary data on a new procedure that utilizes intraprostatic alcohol gel injection to ablate prostatic tissue. The purpose of this study is to evaluate the feasibility of using this gel as a treatment for BPH.

A total of 65 patients with lower urinary tract symptoms (LUTS) due to BPH were treated with intraprostatic injections of alcohol gel. The gel is composed of 97% denatured alcohol and a patented polymer to cause viscosity. Three different methods of injection were utilized: transrectal (TR) injections (8), transurethral (TU) injections (36), and transperineal (TP) injections guided by biplaned ultrasound (21). Each method provided easy access to the center of the prostate, where a volume of gel, approximately 20–30% of the prostatic volume, was injected. Follow-up was based on changes in peak urinary flow (Qmax), IPSS scores, quality of life scores (QoL), adverse effects, and failures. Data are available at 3 and 12 months.

The procedure was well tolerated with only local or no anesthesia in the TR and TP groups; the TU group received spinal anesthesia. All groups showed statistically significant (*p* < 0.0001) improvements in Qmax, IPSS, and QoL. The mean amount of gel injected was 8.05 ml, representing 21.56% of the prostatic volume. Qmax increased from a baseline mean of 8.50 to 12.01 ml/s at 3 months, and to 11.29 ml/s at 12 months. IPSS scores improved from a baseline mean of 21.12 to 10.00 at 3 months, and to 11.84 at 12 months. QoL scores were only available for 55 patients. QoL scores improved from a baseline of 3.93 to 1.98 at 3 months, and to 2.18 at 12 months. No extraprostatic injury or adverse effects were reported due to treatment.

This preliminary study presents significant results showing that intraprostatic injection of alcohol gel could be an option for the treatment of BPH and LUTS. The viscosity of the gel allows for accurate imaging under ultrasound, no run back along the needle allowing for multiple methods of delivery, and the gel does not spread to extraprostatic tissue. This new technique could provide a simple and possibly less expensive clinic procedure for treating BPH, and warrants further study.

## INTRODUCTION

Benign prostatic hyperplasia (BPH) affects 80% of men over the age of 75, and 55% of men over the age of 60, making BPH one of the most common diseases ailing men[1]. During the last decade, three categories of treatment have developed to combat BPH: medications, office procedures, and surgeries. Each type of treatment path should be available to the patient. Medications include alpha blockers, 5 alpha reductase inhibitors, or a combination. Promising results have been found using medications to treat early cases, but medications are usually less successful in treating more developed cases of BPH than office and surgical procedures[2]. Surgical procedures produce the most consistent and durable results of the three categories by removing the obstructive prostatic tissue. However, surgical treatments require a form of general anesthesia, an operating room, hospitalization for recovery, and produce more adverse effects[1].

The office procedure is performed in the physician's office with only local anesthesia; most patients should be able to walk out of the office after completion, usually less than 60 min. The results from office procedures are expected to be better than medical treatments since they directly affect the obstructive tissue and, in some cases, can be comparable to surgical procedures[3]. Currently, there are multiple techniques used in office settings, including transurethral microwave thermotherapy (TUMT), interstial laser coagulation (ILC), transurethral needle ablation (TUNA), and water-induced thermotherapy (WIT). These devices all use a costly machine and a disposable fiber or catheter to generate heat-induced coagulation necrosis and alpha-receptor changes within the prostatic tissue. In most parts of the world, the costs debilitate the use of these office procedures.

To minimize the cost and complexities of office procedures, the development of a simple method to cause coagulation or hemorrhagic necrosis in the prostate has been explored. A simple injection of a chemical into the prostate carries the possibilities of creating an effective in-office treatment for BPH. Denatured alcohol has been used as an ablative agent in the liver and other organs for some time and has been recently used to ablate the prostate[4,5,6,7,8,9]. Studies show promising clinical results, but the liquid alcohol is difficult to control, making accurate placement problematic. Loss of bladder control has occurred due to the spreading of fluid liquid alcohol[4,10].

To utilize the ablative properties of denatured alcohol, a gel containing 97% or greater alcohol has been developed. The viscosity of the alcohol gel minimizes the control problems found with liquid alcohol. The gel's viscosity appears to limit the back flow of the gel along the needle, allowing prostatic injection from the urethra, perineum, or even transrectally. The viscous gel is visible under transrectal ultrasound (TRUS), due to microbubbles inherent in the formulation within the gel. In the TR and TP (transperineal) methods, TRUS allows accurate placement of the needle in the transitional zone. Since the gel is hyperechoic, the TRUS helps to visualize the placement of the ablative agent from base to apex. Cystoscopic visualization is used in the TU method with the depth of penetration measured by the delivery device. In any method of delivery, the gel can even be injected into the median lobe.

This preliminary study represents the clinical results from the first 65 patients with lower urinary tract symptoms (LUTS) due to BPH treated with alcohol gel, including 12-month follow-up. It should be noted that these are the first patients treated, and represent initial attempts to understand the amount of alcohol and placement of the injected gel into the prostate.

## MATERIALS AND METHODS

An IRB-approved study was performed at two institutions: the University of Campinas, Campinas, Brazil and the University of Chile, Santiago, Chile. Enrolled were 65 Latino men, mean age 66 years (±5.42 years, range 52–78 years), with diagnosed BPH and LUTS and scheduled for surgery. Mean prostate size was 37.33 ml (±17.26 ml). Each patient was screened to have LUTS with urinary flow under 17 ml/s and IPSS score greater than 10. Patients had not received other treatments for BPH, including medication.

Three methods of injection were used — TU, TP, and TR — with the decision made by each institution. The action of the chemical is not affected by the course of injection, and each method allows easy access to the center of the transitional zone. The gel is composed of 97% denatured alcohol and a patented polymer to cause viscosity. The ideal volume of gel injected was approximately 20–30% of the prostatic volume. This is similar to the ethanol injection studies[11]. Needle placement was confirmed by TRUS for every patient in the TR and TP group. Cystoscopic visualization of the needle was used in the TU group. It was found to be difficult to visualize the needle in the TU method with TRUS.

Thirty-six (36) patients received TU injection using the *Prostaject* device developed by Injectx (San Jose, CA). This device fits at the bridge of a cystoscope and allows visualization of the needle in the prostatic urethra. This technique is similar to those used with liquid alcohol, and is describe elsewhere[4].

Twenty-one (21) patients received TP injections through an 18- or 20-gauge needle. This procedure was performed using biplane ultrasound to confirm the position of the tip of the needle from base to apex of the transitional zone (see Fig. 1).

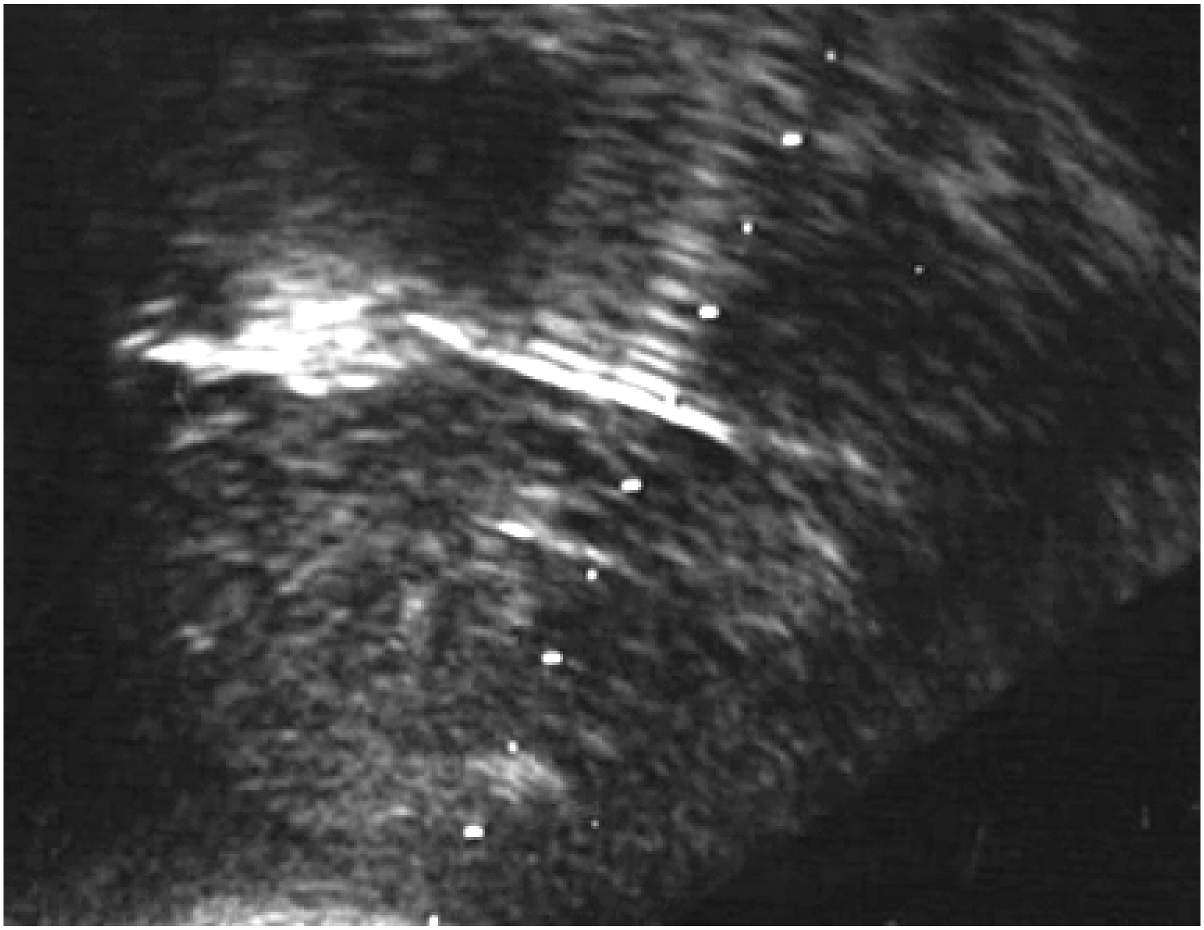

**FIGURE 1**. Sagital view of the prostate with TP needle placement and early injection of alcohol gel.

Eight (8) patients received TR injections through a 20-gauge needle in a procedure similar to TR needle biopsy. These patients lay on their left sides in a modified curled position while the needle was positioned into the transitional zones by a needle guide and TRUS probe (see Fig. 2).

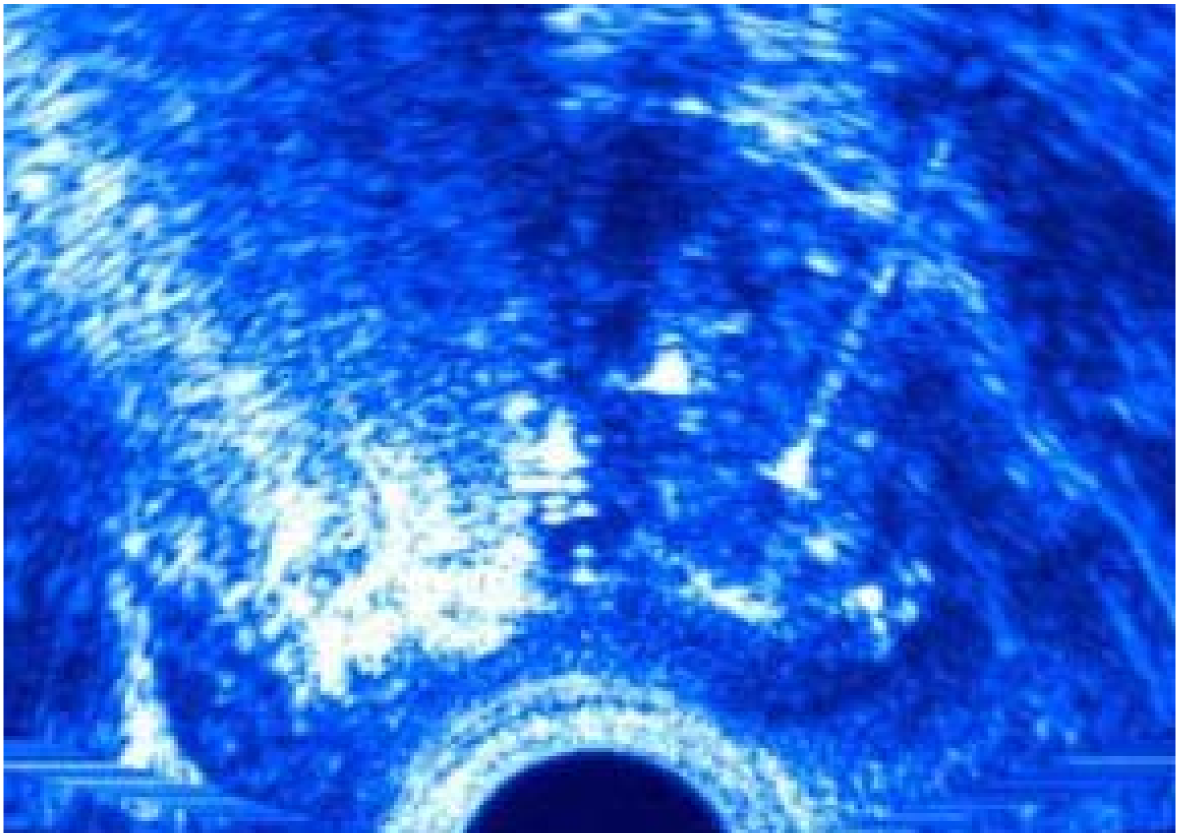

**FIGURE 2**. Transverse view of TR alcohol gel injection of the right transitional zone of the prostate and visualized needle placement in the left transitional zone preparatory to second injection.

Patients were observed for several hours after the procedure and a catheter was placed for 1–4 days. The patients receiving TR injections had the catheter removed after 24 h without need for reinsertion. The other patients had catheters placed for 1–4 days depending on the institution. No trial to evaluate the need and duration of catheterization was conducted in this preliminary study.

Follow-up was based on changes of peak urinary flow rates (Qmax), IPSS scores, quality of life scores (QoL), adverse effects, and failures. Data are available at 3 months and 12 months.

Statistical analysis was performed using JMP software package. Significance was measured by paired t-tests with a 95% ($p < 0.05$) confidence interval.

## RESULTS

The procedure was performed in all TR and TU patients with only local or no anesthesia with most patients in the TU group receiving spinal anesthesia as decided by the treating physician. All treatments, regardless of injection method, produced significant improvements in Qmax, IPSS scores, and QoL (see Table 1). The mean volume of alcohol gel injected into each patient was 8.05 ml (±3.74 ml), which represents 21.56% (range 10.5–37.5%) of the mean prostatic volume.

**TABLE 1**
**Mean Baseline and Follow-Up Data for Alcohol Gel Injection Treatment for LUTS Related to BPH***

| | Baseline | 3 Month | 12 Month |
|---|---|---|---|
| Qmax (ml/s) | 8.50 ± 2.83 (n = 65) | 12.01 ± 4.05 (n = 63) | 11.29 ± 3.68 (n = 57) |
| IPSS | 21.12 ± 5.50 (n = 65) | 10.00 ± 6.64 (n = 63) | 11.84 ± 6.87 (n = 57) |
| QoL | 3.93 ± 1.00 (n = 55) | 1.98 ± 1.28 (n = 52) | 2.18 ± 1.29 (n = 50) |

* All results significant ($p < 0.05$).

Peak flow rates improved significantly from a baseline mean of 8.50 ml/s (±2.83 ml/s, range 1.7–17 ml/s) to 12.01 ml/s (± 4.05, range 3.0–23.7 ml/s, n = 63, $p < 0.0001$) at 3 months and 11.29 ml/s (±3.68 ml/s, range 2.0–23.5 ml/s, n = 57, $p < 0.0001$) at 12 months. Data for each subgroup (TU, TP, and TR) are found in Table 2.

**TABLE 2**
**Mean Baseline and Follow-Up Data for Subgroups (TU, TP, and TR) for Alcohol Gel Injection Treatment for LUTS Related to BPH***

| | Baseline | 3 Month | 12 Month |
|---|---|---|---|
| Qmax (ml/s) | | | |
| TU | 8.22 ± 2.27 (n = 36) | 11.56 ± 3.05 (n = 36) | 10.63 ± 2.69 (n = 32) |
| TP | 9.73 ± 3.62 (n = 21) | 11.62 ± 4.99 (n = 21)** | 11.68 ± 4.44 (n = 18) |
| TR | 6.53 ± 0.67 (n = 8) | 16.08 ± 4.01 (n = 6) | 13.31 ± 5.08 (n = 7) |
| IPSS | | | |
| TU | 19.33 ± 4.64(n = 36) | 8.36 ± 5.57 (n = 36) | 10.91 ± 7.31 (n = 32) |
| TP | 23.90 ± 6.47 (n = 21) | 13.71 ± 7.68 (n = 21) | 13.17 ± 7.09 (n = 18) |
| TR | 21.88 ± 3.04 (n = 8) | 6.83 ± 2.23 (n = 6) | 12.71 ± 3.30 (n = 7) |
| QoL | | | |
| TU | 3.50 ± 0.81 (n = 36) | 1.67 ± 1.01 (n = 36) | 2.09 ± 1.42 (n = 32) |
| TP | 4.74 ± 0.81 (n = 19) | 2.69 ± 1.54 (n = 16) | 2.33 ± 1.03 (n = 18) |
| TR | NA | NA | NA |

* All results significant ($p < 0.05$).

** Not statistically significant ($p = 0.1407$).

IPSS scores improved significantly from a baseline mean of 21.12 (±5.50, range 10–35) to 10.00 (±6.64, range 2–33, n = 63, $p < 0.0001$) at 3 months and 11.84 (±6.87, range 2–32, n = 57, $p < 0.0001$) at 12 months. For IPSS data for each subgroup, see Table 2.

QoL scores were only available for 55 patients from the TU and TP subgroups. These data were not collected for the TR group. The baseline mean QoL score was 3.93 (±1.00), which improved to 1.98 (±1.28, n = 52, $p < 0.0001$) at 3 months and 2.18 (±1.29, n = 50, $p < 0.0001$) at 12 months. For QoL data for each subgroup, see Table 2.

Patients in the TU group all experienced minor hematuria while the catheter was in. No rectal fistula or rectal injury was noted in all groups. No injury to the bladder or any other structure outside of the prostate was noted. No incontinence was found post injection. Three patients were lost to follow-up and one died of unrelated causes. Four patients had subsequent TU resection of the prostate (TURP) during the treatment period (one at <3 months, one at 4 months, and two at 6 months) due to inadequate relief of LUTS.

## DISCUSSION

This preliminary study presents a new potential method for treating LUTS due to BPH. The treatment can be performed by TU and TP methods accurately under TRUS and under cystoscopic visualization by TU method. This treatment modality does not require any expensive capital equipment, thus possibly being available to physicians in most parts of the world.

The results show consistent improvement over 12 months, and while not as good as some other minimally invasive office-based techniques, alcohol gel injection does warrant further investigation and consideration. After 12 months of follow-up, peak flow rates increased 32.8%, IPSS improved by 43.9%, and QoL scores improved by 44.5%. The results show significant improvements in LUTS, but represent only preliminary findings based on a developing technique.

The results are comparable to those of other studies using liquid alcohol[4], but the viscosity of the gel prevents the ablative agent from spreading into undesired areas and allows for perineal and TR approaches. The alcohol gel has been imaged with MRIs and confirmed by pathology to have stayed in the intended target of the transitional zones. This is an advantage over other chemical injections using liquid mediums. Tissue death is caused by hemorrhagic necrosis proven by pathological specimens[12].

Future studies need to determine the best method of injection, the most effective amount of gel to be injected, and where injections should be made within the prostate.

## CONCLUSION

Alcohol gel injection of the prostate for the relief of LUTS due to BPH is possibly a new viable option and warrants further investigation. This preliminary study presents results that show significant improvement in peak flow rate (Qmax), IPSS, and QoL scores at 12 months. The alcohol gel is easy to use and most likely less expensive as compared to other minimally invasive treatments. The viscosity of the gel allows for accurate placement and imaging. No other office treatment has the benefit of imaging to guide and control the amount of treatment. Future studies will determine the most effective manner of using alcohol gel to ablate prostatic tissue and relieve LUTS due to BPH.
